# Characterization of methicillin-susceptible and -resistant staphylococci in the clinical setting: a multicentre study in Nigeria

**DOI:** 10.1186/1471-2334-12-286

**Published:** 2012-11-02

**Authors:** Adebayo Shittu, Omotayo Oyedara, Fadekemi Abegunrin, Kenneth Okon, Adeola Raji, Samuel Taiwo, Folasade Ogunsola, Kenneth Onyedibe, Gay Elisha

**Affiliations:** 1 Department of Microbiology, Obafemi Awolowo University, Ile-Ife, Nigeria; 2 Division of Medical Microbiology, University of Cape Town, Cape Town, Republic of South Africa; 3 Department of Biological Sciences, College of Science, Engineering and Technology, Osun State University, Osogbo, Nigeria; 4 Department of Medical Microbiology, University of Maiduguri Teaching Hospital, Maiduguri, Nigeria; 5 Department of Medical Microbiology and Parasitology, Lagos State University Teaching Hospital, Lagos, Nigeria; 6 Department of Medical Microbiology and Parasitology, Ladoke Akintola University of Technology Teaching Hospital, Ogbomosho, Nigeria; 7 Department of Medical Microbiology and Parasitology, College of Medicine, University of Lagos, Lagos, Nigeria; 8 Department of Medical Microbiology and Parasitology, Jos University Teaching Hospital, Jos, Nigeria

**Keywords:** Coagulase negative staphylococci, *Staphylococcus aureus*, Multiresistance, *mecA* gene, *Staphylococcus haemolyticus*, *Staphylococcus sciuri*, Panton Valentine Leukocidin, SCC*mec* typing, ST152, Nigeria

## Abstract

**Background:**

The staphylococci are implicated in a variety of human infections; however, many clinical microbiology laboratories in Nigeria do not identify staphylococci (in particular coagulase negative staphylococci - CNS) to the species level. Moreover, data from multi-centre assessment on antibiotic resistance and epidemiology of the staphylococci are not available in Nigeria. This study investigated 91 non-duplicate staphylococcal isolates obtained from the microbiology laboratories of eight hospitals in Nigeria during the period January to April 2010.

**Methods:**

Identification and antibiotic susceptibility testing was performed using the VITEK 2 system, detection of resistance genes by PCR, and molecular characterization was determined by SCC*mec* typing, *spa* and multilocus sequence typing (MLST).

**Results:**

All the isolates were susceptible to mupirocin, tigecycline, vancomycin and linezolid, but 72.5% of CNS and 82.3% of *Staphylococcus aureus* were resistant to cotrimoxazole, while multiresistance was observed in 37 of the 40 CNS isolates. Untypeable SCC*mec* types (*ccrC*/Class A *mec* and *ccr*-negative/Class C2 *mec* gene complex) in two methicillin-resistant *S. aureus* (MRSA) were identified. Additionally, *ccr*-negative/Class A *mec* and *ccr* type 4/Class C2 *mec* gene complex was detected in one isolate each of *S. sciuri* and *S. haemolyticus*, respectively. The *S. aureus* isolates were classified into 21 *spa* types including two new types (t8987, t9008) among the methicillin-susceptible *S. aureus* (MSSA) isolates. Two (CC8-SCC*mec*non-typeable and CC88-SCC*mec* IV) and four (CC8-SCC*mec* III/IV/V; CC30-SCC*mec* II/III; CC88-SCC*mec* IV; and ST152-SCC*mec*non-typeable) MRSA clones were identified in Maiduguri (North-East Nigeria) and South-West Nigeria, respectively. The proportion of Panton-Valentine leukocidin (PVL)-positive MSSA was high (44.4%) and 56.3% of these strains were associated with sequence type (ST) 152.

**Conclusions:**

The identification of multiresistant *mecA* positive *S. haemolyticus* and *S. sciuri* from clinical samples indicates that characterization of CNS is important in providing information on their diversity and importance in Nigeria. There is the need to develop new SCC*mec* classification methods for non-typeable methicillin-resistant staphylococci, and to curtail the spread and establishment of the *S. aureus* ST152 clone in Nigeria. The study presents the first report of a PVL-positive ST152-SCC*mec*nontypeable MRSA and SCC*mec* typing of methicillin-resistant CNS in Nigeria.

## Background

The staphylococci are part of the normal cutaneous ecosystem, but they are also opportunistic and invasive pathogens that cause a variety of human infections. *Staphylococcus aureus* is the most important human pathogen in this genus accounting for a high proportion of severe infections in hospitals and outpatient medical care [1-3]. Although the clinical relevance of coagulase negative staphylococci (CNS) is still controversial, patients at risk of CNS infections include neonates, those with intravascular catheters, prosthetic devices, postoperative sternal wound infections and immunocompromised hosts [4-7]. The remarkable ability of *S. aureus* and CNS to acquire antibiotic resistance limits therapeutic options, and morbidity and mortality rates of staphylococcal infections have increased the financial burden on health care systems worldwide [8-14].

Although there are reports on the characterization of staphylococci in Nigeria [15-18], many of the studies were limited to antibiotic susceptibility profiles. Moreover, one geographical region or health care institution was investigated, and CNS were not identified to species level. Only a few studies have investigated the molecular epidemiology and diversity of this group of organisms [19-23], however, they were not multi-centre surveys on *S. aureus* and CNS. This work describes a multi-centre study which was conducted in order to provide clinicians and infection control practitioners with information on antibiotic resistance profiles, molecular characteristics of coagulase positive and negative staphylococci, and to alert clinicians to the emergence of new clones in health care institutions (HCIs) in Nigeria.

## Methods

### Isolation and identification of staphylococcal isolatess

**Figure 1:**
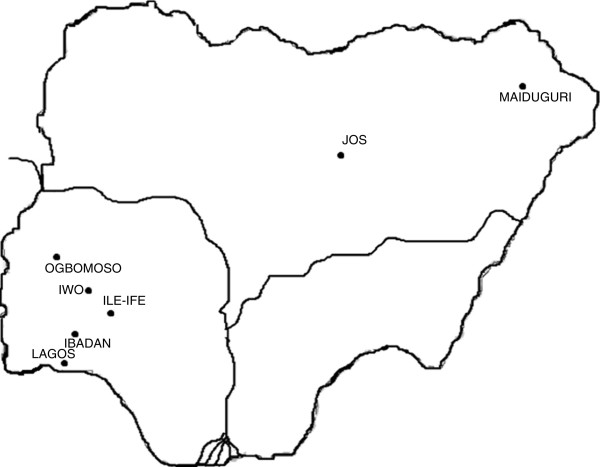
Map indicating the location of the health care institutions in Nigeria.

The study commenced with the request for non-duplicate staphylococcal isolates obtained from clinical samples processed in the microbiology laboratories of eight health care institutions in Ile-Ife, Ogbomosho, Iwo, Lagos (two centres) and Ibadan in South-West Nigeria; Jos in North-Central Nigeria, and Maiduguri in North-East Nigeria (Figure 1) during the period January to April, 2010. In this study, only staphylococcal isolates were analyzed and human subjects, clinical samples or human data were not investigated. All the hospitals, except the one located in Iwo, were tertiary health care institutions, each of which were at least 500-bed facilities providing medical care to about 500,000 to one million people. Preliminary verification as staphylococci was based on culture characteristics on blood agar, catalase, coagulase and DNase tests, while identification to species level was performed at the National Health Laboratory Service (NHLS), Groote Schuur Hospital, Cape Town, South Africa using the VITEK 2 system (bioMérieux, France) with the Gram-Positive Identification Card.

### Antibiotic susceptibility testing

Antibiotic susceptibility for penicillin G, oxacillin, teicoplanin, vancomycin, gentamicin, tetracycline, ciprofloxacin, moxifloxacin, trimethoprim/sulfamethoxazole (cotrimoxazole), fusidic acid, erythromycin, clindamycin, rifampicin, mupirocin, linezolid and tigecycline was determined by the broth microdilution method (VITEK 2 system, AST 603 cards, bioMérieux). *S. aureus* ATCC 29213 was the control strain and the VITEK 2 minimum inhibitory concentration (MIC) results were interpreted using the Advanced Expert System of the VITEK 2. Multiresistance was defined as resistance to at least three classes of antibiotics.

### DNA extraction

Before DNA extraction, 5–8 colonies from an 18–24 h old culture on boiled blood agar was pretreated with lysostaphin (Sigma) (20 μg/ml for *S. aureus*; 50μg/ml for CNS) in 20 mM Tris-Cl, 2mM EDTA at 37°C for 30 min. The cells were harvested and DNA was isolated using a DNeasy tissue kit (Qiagen, Hilden, Germany).

### PCR detection of the *tuf* gene

Phenotypic identification of the *S. aureus* isolates was confirmed by detection of the *tuf* gene as described previously [24]. The PCR products were detected by gel electrophoresis using 2% w/v agarose (Seakem, Whittaker, USA) and run in 1X TAE buffer (pH 8.3) for 1 h at 100 V. Thereafter, the gel was stained with ethidium bromide, visualized under UV light and photographed using a SynGene Bioimaging System.

### PCR detection of the *dfrA* gene

The presence of the *dfrA* gene encoding trimethoprim resistance was investigated using the primer pairs: dfr-AF: AAT AGA CGT AAC GTC GTA CT; dfr-AR: AAG AAT GTA TGC GGT ATA GT and subsequent detection of a 289 bp product [25]. The PCR conditions were as follows: Initial denaturation at 95°C for 3min, followed by 30 cycles of amplification at 95°C for 30s, annealing at 55°C for 30s, extension at 72°C for 30s and final extension at 72°C for 1min.

### SCC*mec* typing

SCC*mec* typing was performed according to a previously published protocol [26] using the control strains *S. aureus* COL (type I), N315 (type II), ANS46 (type III), MW2 (type IV), WIS (type V) and HDE288 (type VI). For non-typeable SCC*mec* elements, the investigation of *mec* complexes and *ccr* allotypes as described by Kondo et al. [27] was carried out on representative methicillin-resistant *S. aureus* (MRSA) and CNS isolates based on their *spa*-MLST profile and location of the health care institutions, respectively. The profiles obtained were characterized and defined according to the current nomenclature used for MRSA [28].

### Detection of the PVL gene

The presence of the PVL gene was determined by PCR using primers luk-PV-1 and luk-PV-2 [29].

### Genotyping

Typing of *S. aureus* was based on sequencing of the hypervariable region of the protein A gene (*spa*). The *spa* types were assigned using the Ridom StaphType (Ridom GmbH, Würzburg, Germany, version 1.5.21) [30]. Multilocus sequence typing (MLST) was carried out according to the protocols described previously [31] on a representative isolate for the predominant and new *spa* types. PCR products were purified and sequenced at the Central Analytical Facility at the University of Stellenbosch, South Africa. Allelic profiles and sequence types (STs) were assigned using the MLST *S. aureus* database (http://www.mlst.net). Isolates that were not typed by MLST were assigned STs based on the Based Upon Repeat Pattern (BURP) via the Ridom StaphType software.

## Results

### Identification and source of the staphylococcal isolates

Preliminary verification of isolates obtained from the microbiology laboratories in eight HCIs in Nigeria identified 91 staphylococcal isolates (51 *S. aureus* and 40 CNS) and the characteristics of each isolate are presented in Tables 1, 2 and 3. The clinical *S. aureus* isolates were recovered from wounds, skin and soft tissue infections (SSI), osteomyelitis and burns (36.4%), genitourinary tract infection (GTI)/infertility (18.2%), septicaemia (15.9%), urinary tract infection (UTI) (6.8%), otitis media (6.8%), bronchitis (4.5%), and 11.4% were classified as other infections (Tables 1 and 2). Six species accounted for the CNS isolates: *S. haemolyticus* (n=21), *S. sciuri* (n=9), *S. saprophyticus* (n=5), *S. warneri* (n=3), *S. epidermidis* (n=1) and *S. hominis* (n=1), which were distributed across four HCIs located in Ogbomosho, Lagos (designated as Lagos2), Jos and Maiduguri (Table 3). Most of the *S. haemolyticus* isolates were from the centres in Lagos2, Jos and Maiduguri, and 47.6% of the isolates were obtained from septicaemia, 14.3% from wound infections, 9.5% each from GTI, UTI, ocular-related infections, and those categorised as other infections (Table 3).

**Table 1:** Characterization of MRSA from Nigeria by antibiotic resistance pattern, detection of genes and molecular typing

**Isolate No**	**Location**	**Sample Or Clinical Diagnosis**	**Antibiogram**	***tuf *gene**	***mecA *gene**	***dfrA *gene**	**SCC*mec *typing**	**PVL gene**	***spa *type**	**MLST (ST)**	**Clonal Complex (CC)**
50	Lagos1	Burns	PEN, OXA, GEN, CIP, MOX, ERY, CLI*, TET, COT, RIF	+	+	+	III	-	t037	ST241	CC8
2496^ψ^	Maiduguri	GTI	PEN, OXA, GEN, CIP, ERY, CLI, TET, COT	+	+	-	NT/UNT	-	t037**		
2589	Maiduguri	GTI	PEN, OXA, GEN, CIP, ERY, CLI, TET, COT	+	+	-	NT	-	t037		
60^ψ^	Lagos1	ASOM	PEN, OXA, TET, COT	+	+	-	NT/V	-	t064**	ST8	CC8
69^¶^	Lagos1	Nasal swab/Screening	PEN, OXA, GEN, TET, COT	+	+	-	NT	-	t064		
T39sB	Iwo	HIV/AIDS	PEN, OXA, TET, COT	+	+	-	NT	-	t064		
T6530	Ile-Ife	Osteomyelitis	PEN, OXA, GEN, CIP, TET, COT	+	+	-	IV	-	t064		
TU32	Ibadan	Wound Infection	PEN, OXA, CIP, TET, COT	+	+	-	NT	-	t064		
TU3^ψ^	Ibadan	Wound Infection	PEN, OXA, CIP, TET, COT	+	+	-	NT/V	-	t064		
T5056	Ile-Ife	Bronchitis	PEN, OXA, GEN, CIP, ERY, CLI*, TET, COT, FUS, RIF	+	+	-	III	-	t074	ST37	CC30
TD17	Ile-Ife	Bronchitis	PEN, OXA, ERY, CLI	+	+	ND	II	-	t007	ST39	
TN45	Ile-Ife	Wound Infection	PEN, OXA, TET	+	+	ND	IV	-	t729	ST88	CC88
003B	Maiduguri	Septicaemia	PEN, OXA, TET, COT	+	+	+	IV	-	t1603		
008	Maiduguri	Septicaemia	PEN, OXA, COT	+	+	+	IV	-	t1603		
T5843^ψ^	Ile-Ife	Osteomyelitis	PEN, OXA, TET, COT	+	+	-	NT/UNT	+	t4690**	ST152	singleton

KEY:

^ψ^: Representative MRSA for SCC*mec* typing determined by Kondo *et al*. (2007).

¶Nasal samples of health care workers (HCWs) in one of the hospitals in Lagos (designated as Lagos1).

Geographical region (Nigeria): South-West (Ile-Ife, Ibadan, Iwo and Lagos); North-East (Maiduguri); North Central (Jos).

*GTI*: Genital Tract Infection; ASOM: Acute suppurative otitis media.

Antibiotics - PEN: Penicillin; OXA: Oxacillin; GEN: Gentamicin; CIP: Ciprofloxacin; MOX: Moxifloxacin; ERY: Erythromycin; CLI: Clindamycin; TET: Tetracycline; FUS: Fusidic acid; RIF: Rifampicin; COT: Cotrimoxazole; *: Inducible resistance (clindamycin).

*UNT:* Untypeable SCC*mec* types; NT: Non-typeable; ND: Not determined.

Isolates with *spa* type t007, t074, t729 and t1603 were assigned STs based on the Based Upon Repeat Pattern (BURP) via the Ridom StaphType software.

**: MLST on representative strains.

**Table 2:** Characterization of MSSA from Nigeria by antibiotic resistance pattern, detection of genes and molecular typing

**Isolate No**	**Location**	**Sample Or Clinical Diagnosis**	**Antibiotype**	***tuf *gene**	***dfrA *gene**	**PVL gene**	***spa *type**	**MLST (ST)**	**Clonal Complex (CC)**

1229	Maiduguri	GTI	TET	+	ND	-	t311	ST5	CC5
652	Maiduguri	Not available	PEN, COT	+	-	+	t311		
47	Lagos1	Leg Ulcer	PEN, TET, CIP, MOX, COT	+	-	-	t064		CC8
54	Lagos1	SLE	PEN, TET, COT, CIP	+	-	-	t064		
49^¶^	Lagos1	Nasal swab/screening	PEN, COT	+	-	-	t2331**	ST8	
10	Ogbomosho	SSI	PEN	+	ND	+	t084		CC15
62	Lagos1	UTI	COT	+	-	-	t084		
076	Maiduguri	Not available	PEN, TET, COT	+	-	-	t084		
373	Maiduguri	UTI	COT, TET	+	-	-	t084**	ST15	
1253	Maiduguri	SSI	PEN, TET, COT	+	-	-	t084		
2290	Maiduguri	SSI	PEN, TET, COT	+	-	-	t084		
2696	Maiduguri	Semen (Infertility)	PEN, COT	+	-	-	t084		
1038	Maiduguri	SSI	PEN, COT	+	-	+	t2216		
2478	Maiduguri	UTI	PEN, TET, COT	+	-	-	t2216		
6B	Ogbomosho	Septicaemia	COT	+	-	+	t318	ST30	CC30
51^¶^	Lagos1	Nasal swab/screening	PEN, COT	+	-	-	t318		
1059	Maiduguri	GTI	COT	+	-	-	t318		
2578	Maiduguri	GTI	PEN, TET, COT	+	-	+	t934	ST80	CC80
56	Lagos1	Otitis media	PEN, COT	+	-	-	t159	ST121	CC121
67^¶^	Lagos1	Nasal swab/screening	PEN, TET, COT	+	-	+	t2304		
68^¶^	Lagos1	Nasal swab/screening	PEN, TET, COT	+	-	+	t2304		
006	Maiduguri	Septicaemia	PEN, TET, COT	+	-	+	t355**	ST152	singleton
0017	Maiduguri	Septicaemia	PEN, TET, COT	+	-	+	t355		
1097	Maiduguri	SSI	PEN, TET, COT	+	-	+	t355		
2365	Maiduguri	Semen (Infertility)	PEN, COT	+	-	+	t355		
259	Maiduguri	Semen (Infertility)	PEN, COT	+	-	+	t355		
604	Maiduguri	SSI	PEN, TET, COT	+	-	-	t355		
642	Maiduguri	SSI	PEN, TET	+	ND	+	t9008**		
829	Maiduguri	SSI	PEN, TET	+	ND	-	**t9008**		
J11	Jos	Septicaemia	PEN, TET, COT	+	-	+	t355		
J13	Jos	Septicaemia	PEN, TET, COT	+	-	+	t355		
61	Lagos1	SSI	TET, COT	+	-	+	t8987**		
64^¶^	Lagos1	Nasal swab/screening	PEN	+	ND	-	t1510	ST508	CC45
55	Lagos1	CSOM	PEN	+	ND	-	t8223	ND	CC45
57	Lagos1	Haematoma	Susceptible to all antibiotics	+	ND	-	t635	ND	ND
70^¶^	Lagos1	Nasal swab/screening	PEN, TET, COT	+	-	-	NT	ND	ND

KEY:

^¶^: Nasal samples of health care workers (HCWs) in one of the hospitals in Lagos (designated as Lagos1).

*GTI:* Genital Tract Infection; SLE: systemic lupus erythematosus; SSI: Skin and soft tissue infection; UTI: Urinary Tract Infection; CSOM: Chronic suppurative otitis media.

Antibiotics - PEN: Penicillin; TET: Tetracycline; CIP: Ciprofloxacin; MOX: Moxifloxacin; COT: Cotrimoxazole.

*ND:* Not determined; NT: Non-typeable.

*Spa* types (bold) are new types; Isolates with *spa* type t064, t159, t311, t318, t934, 1510, t2216 and t2304 were assigned STs based on the Based Upon Repeat Pattern (BURP) via the Ridom StaphType software. **: MLST on representative strains.

**Table 3:** **Identification and Characterization of CNS by antibiotic resistance pattern, *mecA *gene detection and SCC*mec *typing**

**Isolate No**	**Location**	**Sample/Clinical Condition**	**VITEK 2 Identification**	**Antibiogram**	***mecA *gene**	**SCC*mec *Typing**
2	Ogbomosho	Wound Infection	*S. saprophyticus*	PEN, TET, COT, FUS	-	ND
4	Ogbomosho	UTI	*S. saprophyticus*	PEN, TET, COT, FUS	-	ND
7	Ogbomosho	Osteomyelitis	*S. saprophyticus*	PEN, TET, COT, FUS	-	ND
13B	Ogbomosho	UTI	*S. saprophyticus*	PEN, COT, FUS	-	ND
14^ψ^	Ogbomosho	Septicaemia	*S. sciuri*	PEN, OXA, GEN, CIP, FUS, MOX	+	NT/UNT
16	Ogbomosho	SSI	*S. warneri*	PEN, TET, COT, FUS	-	ND
16B	Ogbomosho	Osteomyelitis	*S. warneri*	TET, COT, FUS	-	ND
18	Ogbomosho	SSI	*S. saprophyticus*	PEN, TET, COT, FUS	-	ND
66	Lagos2	Wound Infection	*S. sciuri*	PEN, OXA, GEN, CIP, FUS	+	NT
L2^ψ^	Lagos2	Septicaemia	*S. haemolyticus*	PEN, OXA, TET, COT	+	NT/V
L5	Lagos2	Eye swab	*S. haemolyticus*	PEN, OXA, CIP, MOX, TET, COT	+	NT
L6	Lagos2	Septicaemia	*S. haemolyticus*	PEN, OXA, GEN, CIP, ERY, TET, RIF, COT	+	NT
L7	Lagos2	Septicaemia	*S. haemolyticus*	PEN, OXA, GEN, CIP, MOX, ERY, TET, COT	+	NT
L8	Lagos2	Septicaemia	*S. haemolyticus*	PEN, OXA, GEN, CIP, ERY, CLI*, TET, COT	+	NT
L9	Lagos2	Septicaemia	*S. haemolyticus*	PEN, OXA, GEN, CIP, TET, COT	+	NT
L10	Lagos2	Eye swab	*S. haemolyticus*	PEN, OXA, GEN, CIP, MOX, ERY, TET, COT	+	NT
1103	Maiduguri	Wound Infection	*S. warneri*	PEN, OXA, TET	+	NT
1268	Maiduguri	Wound Infection	*S. haemolyticus*	PEN, OXA, GEN, CIP, MOX, TET, COT	+	NT
024^ψ^	Maiduguri	Wound Infection	*S. haemolyticus*	PEN, OXA, GEN, TET, COT	+	NT/V
2491	Maiduguri	UTI	*S. haemolyticus*	PEN, OXA, GEN, CIP, ERY, TET, COT, TEICOi	+	NT
2412	Maiduguri	Wound Infection	*S. haemolyticus*	PEN, OXA, GEN, CIP, MOX, TET, COT	+	NT
2362 ^ψ^	Maiduguri	GTI	*S. haemolyticus*	PEN, OXA, GEN, CIP, ERY, CLI*, TET, COT	+	NT/UNT
825	Maiduguri	GTI	*S. haemolyticus*	PEN, OXA, GEN, CIP, TET, COT	+	NT
2502	Maiduguri	UTI	*S. haemolyticus*	PEN, OXA, GEN, COT	ND	ND
J2	Jos	Septicaemia	*S. hominis*	PEN, OXA, TET	ND	ND
J5	Jos	Septicaemia	*S. haemolyticus*	PEN, OXA, GEN, CIP, ERY, CLI*, TET, RIF, COT	ND	ND
J6	Jos	Septicaemia	*S. haemolyticus*	PEN, OXA, GEN, CIP, ERY, CLI*, TET, RIF, COT	ND	ND
J10	Jos	Septicaemia	*S. haemolyticus*	PEN, OXA, GEN, CIP, ERY, CLI*, TET, RIF, COT	ND	ND
J14^ψ^	Jos	Septicaemia	*S. haemolyticus*	PEN, OXA, GEN, CIP, ERY, TET, FUS, COT	+	NT/V
J15	Jos	Prostatis	*S. haemolyticus*	PEN, OXA, TET, COT	+	NT
J16	Jos	Otitis media	*S. haemolyticus*	PEN, OXA, GEN, CIP, COT	+	NT
J17B	Jos	Osteomyelitis	*S. epidermidis*	PEN, OXA, COT	+	NT
151	Ogbomosho	Septicaemia	*S. haemolyticus*	PEN, OXA, GEN, CIP, MOX, TET, COT	+	NT
12	Ogbomosho	Conjunctivitis	*S. sciuri*	PEN, OXA, GEN, CIP, FUS	+	NT
19B	Ogbomosho	SSI	*S. sciuri*	PEN, OXA, GEN, CIP, TET, FUS	+	NT
11	Ogbomosho	Wound abscess	*S. sciuri*	PEN, OXA, GEN, CIP, FUS	+	NT
17	Ogbomosho	Wound infection	*S. sciuri*	PEN, OXA, CIP, GEN, FUS	+	NT
11B	Ogbomosho	UTI	*S. sciuri*	PEN, OXA, GEN, CIP, MOX, FUS	+	NT
15	Ogbomosho	UTI	*S. sciuri*	PEN, OXA, GEN, CIP, MOX, FUS	+	NT
6	Ogbomosho	UTI	*S. sciuri*	PEN, OXA, GEN, CIP, FUS	+	NT

KEY:^ψ^: Representative MRCNS for SCC*mec* typing determined by Kondo *et al*. (2007); *UTI:* Urinary Tract Infection; *SSI:* Skin and soft tissue infection; *GTI:* Genital Tract Infection. Antibiotics - *PEN:* Penicillin; *OXA:* Oxacillin; *GEN:* Gentamicin; CIP: Ciprofloxacin; MOX: Moxifloxacin; ERY: Erythromycin*; CLI:* Clindamycin; *TET:* Tetracycline; *FUS:* Fusidic acid; *RIF:* Rifampicin; *COT:* Cotrimoxazole; *TEICO:* Teicoplanin. *: Inducible resistance (clindamycin); *ND:* Not determined; *NT:* Non-typeable; *UNT:* Untypeable SCC*mec* types.

### Antibiotic susceptibility of the staphylococcal isolates and detection of *mecA* and *dfrA* genes

All the staphylococcal isolates were susceptible to mupirocin, tigecycline, vancomycin and linezolid (Table 4). In addition to the antibiotics stated above, the methicillin-susceptible *S. aureus* (MSSA) were susceptible to teicoplanin, gentamicin, erythromycin, clindamycin, fusidic acid and rifampicin. About 94% of MSSA were susceptible to the fluoroquinolones and one isolate was susceptible to all the antibiotics tested. However, 80.6% of MSSA were resistant to cotrimoxazole and penicillin, but all the cotrimoxazole-resistant MSSA were *dfrA* negative. The predominant antibiotype was resistance to penicillin, tetracycline and cotrimoxazole, which was observed in 14 isolates (39%). Resistance to oxacillin was detected in 15 *S. aureus* isolates and confirmed as MRSA by the detection of the *mecA* gene. Of the 15 MRSA isolates, 13 were resistant to cotrimoxazole and tetracycline, while 6 and 7 MRSA were resistant to gentamicin and ciprofloxacin, respectively. Two of the 5 erythromycin-resistant MRSA expressed inducible resistance to clindamycin. Overall, 12 of the 15 MRSA were resistant to at least three classes of antibiotics (Table 1). The trimethoprim resistance gene, *dfrA*, was detected in only 3 MRSA isolates.

**Table 4:** Antibiotic resistance profile of staphylococci from Nigeria

	**Number (%) of isolates resistant among**			
**Antibiotics**	**MSSA**	**MRSA**	***S. haemolyticus***	**Others (CNS)**
	**n=36**	**n=15**	**n=21**	**n=19**
Penicillin	29 (80.6)	15 (100)	21 (100)	18 (94.7)
Oxacillin	0 (0)	15 (100)	21 (100)	12 (63.2)
Teicoplanin	0 (0)	0 (0)	1* (4.8)	0 (0)
Vancomycin	0 (0)	0 (00	0 (0)	0 (0)
Gentamicin	0 (0)	6 (40)	18 (85.7)	9 (47.4)
Tetracycline	21 (58.3)	13 (86.7)	19 (90.5)	9 (47.4)
Ciprofloxacin	2 (5.6)	7 (46.7)	17 (80.9)	9 (47.4)
Moxifloxacin	1 (2.8)	1 (6.7)	6 (28.6)	3 (15.8)
Erythromycin	0 (0)	5 (33.3)	10 (47.6)	0 (0)
Clindamycin	0 (0)	5 (33.3)	5 (23.8)	0 (0)
Fusidic acid	0 (0)	1 (6.7)	1 (4.8)	16 (84.2)
Tigecycline	0 (0)	0 (0)	0 (0)	0 (0)
Mupirocin	0 (0)	0 (0)	0 (0)	0 (0)
Linezolid	0 (0)	0 (0)	0 (0)	0 (0)
Rifampicin	0 (0)	2 (13.3)	4 (19)	0 (0)
Cotrimoxazole	29 (80.6)	13 (86.7)	21 (100)	8 (42.1)

*Intermediate resistance.

All the *S. haemolyticus* isolates were resistant to penicillin, oxacillin and cotrimoxazole, but only one isolate exhibited resistance to fusidic acid. The proportion of *S. haemolyticus* isolates resistant to ciprofloxacin, gentamicin and tetracycline was 80.9%, 85.7% and 90.5%, respectively (Table 4). Furthermore, 5 (50%) of the 10 erythromycin-resistant *S. haemolyticus* isolates expressed inducible resistance to clindamycin, and one isolate showed intermediate resistance to teicoplanin (MIC: 16μg/ml). Multiresistant *S. haemolyticus* isolates were recovered from clinical samples in four HCIs in Lagos2, Maiduguri, Jos and Ogbomosho (Table 3). In contrast, resistance to fusidic acid was common in other species of CNS, and they were susceptible to erythromycin, clindamycin, teicoplanin and rifampicin (Table 4). The *S. sciuri* isolates were resistant to oxacillin and obtained primarily from various clinical samples in Ogbomosho (Table 3). On the whole, multiresistance was observed in 37 of the 40 CNS isolates, and the CNS isolates identified as methicillin-resistant (VITEK) were *mecA* positive.

### SCC*mec* typing

Fifteen MRSA were obtained from the HCIs in Ile-Ife (n=5); Maiduguri (n=4); Lagos1 (n=3); Ibadan (n=2) and Iwo (n=1). Using the multiplex typing method [26], SCC*mec* type IV was identified in two MRSA each from Ile-Ife and Maiduguri, SCC*mec* type III in two MRSA, one each from Ile-Ife and Lagos1, and SCC*mec* type II in one MRSA from Ile-Ife (Table 1). However, 8 of 15 MRSA and 28 methicillin-resistant CNS (MRCNS) were non-typeable. Based on another PCR-based method [27], two of the four representative MRSA and three *S. haemolyticus* possessed the SCC*mec* V element (*ccrC*/Class C2 *mec*). However, untypeable SCC*mec* elements were detected in two MRSA (*ccrC*/Class A *mec* and *ccr*-negative/Class C2 *mec*) and one isolate each of *S. sciuri* and *S. haemolyticus* (*ccr*-negative/Class A *mec* and *ccr* type 4/Class C2 *mec* gene complex) (Tables 1 and 3).

### Detection of PVL gene

One MRSA from Ile-Ife was PVL positive (Table 1). The gene was also detected in 16 MSSA, of which 10 were isolated from patients with SSI and septicaemia (Table 2).

### Molecular typing of MSSA and MRSA

A total of 21 *spa* types were identified and representative *S. aureus* strains assigned as t037, t064, t084, t355, t2331, t4690, and new types t8987 and t9008 were selected for MLST (Tables 1 and 2). In MRSA, seven different *spa* types were identified and isolates with *spa* types t037, t064, and t4690 were assigned ST241, ST8 and ST152, respectively. Overall, the clonal complexes for MRSA were distributed across CC8, CC30, CC88 and ST152 (Table 2). In MSSA, fifteen different *spa* types, including two new types (t8987 and t9008) were identified of which only t064 was identified also in MRSA (Tables 1 and 2). The predominant *spa* types were t355 (8 isolates), t084 (7 isolates) and t318 (3 isolates) and a diversity of *spa* types (t318, t1510, t2304, t2331, *spa* non-typeable) were identified among the MSSA from nasal samples of health care workers (HCWs) (Table 2). MSSA isolates with *spa* type t084 and t2331 were assigned ST15 and ST8 respectively, while ST152 accounted for the new *spa* types (t8987, t9008) and t355. Nine and 11 of the 36 MSSA clustered with ST15 and ST152 respectively (Table 2).

## Discussion

The identification of bacterial isolates to species level is of great importance in the clinical microbiology laboratory to obtain information on the diversity and significance of each species in human infection. Moreover, species identification is a prerequisite for epidemiological studies. Many clinical microbiology laboratories in Nigeria do not identify staphylococci (in particular CNS) to species level, which is important in understanding their variety and clinical relevance. Two species, *S. haemolyticus* and *S. sciuri*, accounted for the majority of the 40 CNS isolates investigated in this study (Table 3). Most of the *S. haemolyticus* isolates were obtained from blood culture samples in HCIs in Lagos2 and Jos, and *S. sciuri* from samples in Ogbomosho (South-West Nigeria). *S. haemolyticus* is regarded as the second most frequently isolated staphylococci from blood cultures [32] and one of the most clinically relevant CNS, particularly in immunocompromised patients [33]. *S. sciuri*, principally found in animal species, and although not considered important from the clinical standpoint, has been associated with infections such as endocarditis [34], urinary tract infection [35] and wound infections [19,36]. Multiresistance to antibiotics was detected in 37 of the 40 CNS isolates, and all the *S. haemolyticus* isolates were resistant to at least three classes of antibiotics. Apart from the possible clinical importance because of its serious impact on the course of infection, multiresistant CNS are a potential source of genes encoding resistance to antibiotics for other staphylococci pathogenic for man. The isolation of multiresistant *mecA* positive *S. haemolyticus* from blood samples (septicaemia) and *S. sciuri* from various clinical materials (Table 3) in some HCIs clearly indicate that they could be of clinical importance in Nigeria. A number of reports have indicated an increase in the resistance of staphylococci to cotrimoxazole in Nigeria [15,18,21,23,37]. In this study, 72.5% of CNS and 82.3% of *S. aureus* isolates were resistant to cotrimoxazole. The antibiotic has wide clinical application, inexpensive, orally administered and available over-the-counter where they are sold with or without prescription in Nigeria. This could possibly explain the high level of staphylococcal resistance observed in this study. Most of the cotrimoxazole-resistant *S. aureus* were *dfrA*-negative; hence more studies are needed to understand the molecular mechanism of resistance in Nigeria.

Methicillin resistance in staphylococci is due to the expression of the *mecA* gene, which mediates penicillin binding protein 2a (PBP2a), a transpeptidase with low affinity for beta-lactams [7,38]. The *mecA* gene is carried by a mobile genetic element (MGE) termed the staphylococcal cassette chromosome (SCC*mec*), and though the *mec* origin remains unknown, it has been suggested that *mecA* CNS may act as potential SCC*mec* donor accounting for the rise of new MRSA clones [39]. Eleven SCC*mec* elements have been described to date: SCC*mec* I-IV [40-42], and V-XI [43-48], but few reports exist on the detection of *mecA* gene and characterization of SCC*mec* types in Nigeria [21-23]. There was excellent correlation between results of antibiotic susceptibility testing (VITEK) and detection of the *mecA* gene for the confirmation of methicillin-resistant staphylococci. However, the MRCNS were non-typeable using the multiplex PCR protocol [26]. Based on the method of Kondo et al. [27], the SCC*mec* V element (*ccrC*/Class C2 *mec*) was identified in representative *S. haemolyticus* isolates, as well as untypeable SCC*mec* elements (*ccr*-negative/Class A *mec* and *ccr* type 4/Class C2 *mec* gene complex) in one representative isolate each of *S. sciuri* and *S. haemolyticus* (Table 3). Recent studies have indicated that SCC*mec* V is the predominant element among methicillin-resistant *S. haemolyticus* isolates [33,49]. To the best of our knowledge, this is the first report on the characterization of SCC*mec* elements in CNS in Nigeria. It is likely that the *mec* gene complex and *ccr* genes in CNS are diverse and distinct, and as indicated in previous reports [7,28], there is the need to develop new classification schemes for non-typeable SCC*mec* types in MRCNS.

Molecular typing of the MRSA isolates indicated that two clones (CC8-SCC*mec*non-typeable and CC88-SCC*mec* IV) existed in Maiduguri, North-East Nigeria, and four clones in South-West Nigeria (CC8-SCC*mec*III/IV/V; CC30-SCC*mec*II/III; CC88-SCC*mec*IV and ST152-SCC*mec*non-typeable) (Table 1). Previous studies on the epidemiology of MRSA in Nigeria identified MRSA clones t064-ST8-CC8 and t037-ST241-CC8 in South-West and North-East Nigeria, respectively [22,23]. A community-associated clone, CC88-MRSA-IV, previously reported in Ibadan, Nigeria [21] was identified from wound and blood samples in Maiduguri and Ile-Ife, however, we were unable to determine whether the isolates were community-associated. Untypeable SCC*mec* types (*ccrC*/Class A *mec*; *ccr*-negative/Class C2 *mec*) were detected in two representative MRSA isolates, of which one was a PVL-positive isolate (t4690-ST152) from a patient with osteomyelitis in Ile-Ife (South-West Nigeria).

The MSSA were assigned mainly to clonal complexes CC5, CC8, CC15, CC30, CC45, CC80 and ST152 (Table 2). A high proportion of MSSA from Maiduguri were grouped in ST15 or ST152 indicating that these clones were successful in North East Nigeria. PVL-encoding genes were detected in 16 MSSA (44.4%) isolates belonging to almost all clonal complexes identified in this study. Moreover, 56% of these strains were associated with ST152 and 10 of 16 (62.5%) PVL positive MSSA isolates were associated with SSI and septicaemia. These observations confirm previous studies that PVL-positive MSSA ST152 are widespread in African countries [50-52] including Nigeria [22,23]. Furthermore, MSSA isolates (ST121) from nasal samples of HCWs were PVL positive. Our data suggests that the sequence types for pandemic PVL-positive MSSA identified in this study did not function as a reservoir for PVL-positive MRSA supporting the argument of Schaumburg et al. [52] that the intimate inter-relation between PVL-positive MSSA and MRSA, which could lead to the emergence and spread of PVL-positive MRSA clones has not been established. However, the detection of an ST152-PVL-positive MRSA in this study suggests that surveillance for this clone is strongly advocated in Nigeria.

## Conclusions

This is the first multi-centre study on the characterization of coagulase positive and negative staphylococci using phenotypic and molecular methods in Nigeria. However, there were a number of limitations in this study. The number of isolates from the HCIs was low and variable which did not allow for comparison of data among the participating centres. Moreover, the diversity of the staphylococci analysed in this study does not represent their distribution patterns in health care institutions in Nigeria. Lastly, we could not conduct full characterization of the *mecA*-positive CNS isolates, and only representative isolates were selected for SCC*mec* typing and MLST. However, this study has highlighted the importance of species identification for CNS, their clinical relevance, and the need for longitudinal multi-centre surveys to provide data on antibiotic resistance and epidemiology of the staphylococci in Nigeria. Characterization of multiresistant *mecA* positive *S. haemolyticus* and *S. sciuri* from clinical samples is important in understanding their epidemiology within hospitals in Nigeria. The identification of untypeable SCC*mec* types in the study will be further evaluated and characterized; however, there is the need to develop new SCC*mec* classification schemes for non-typeable methicillin-resistant staphylococci. There is an urgent need to curtail the spread and establishment of PVL-positive MSSA, in particular, the MSSA ST152 clone, in Nigeria.

## Abbreviations

SCC*mec*: Staphylococcal Cassette Chromosome *mec*; CC: Clonal Complex; PVL: Panton Valentine Leukocidin; ST: Sequence type; CNS: Coagulase negative staphylococci; HCIs: Health Care Institutions; MIC: Minimum Inhibitory Concentration; MLST: Multilocus sequence typing; ASOM: Acute suppurative otitis media; CSOM: Chronic suppurative otitis media; SLE: Systemic lupus erythematosus; GTI: Genitourinary tract infection; UTI: Urinary tract infection; SSI: Skin and soft tissue infection; MSSA: Methicillin-susceptible *Staphylococcus aureus*; MRSA: Methicillin-resistant *Staphylococcus aureus*; MRCNS: Methicillin-resistant coagulase negative staphylococci; NT: Non-typeable; ND: Not determined; UNT: Untypeable; Antibiotics:; PEN: Penicillin; OXA: Oxacillin; TEICO: Teicoplanin; TET: Tetracycline; GEN: Gentamicin; CIP: Ciprofloxacin; MOX: Moxifloxacin; ERY: Erythromycin; CLI: Clindamycin; FUS: Fusidic acid; RIF: Rifampicin; COT: Cotrimoxazole.

## Competing interests

The authors declare that they have no competing interest.

## Authors’ contributions

AOS, KO, AR, ST, FO, KO and GE participated in the design of the study. OO and FA performed the preliminary identification of the isolates. AOS carried out the molecular characterization of the isolates. All authors read and approved the final manuscript.

## Pre-publication history

The pre-publication history for this paper can be accessed here:

http://www.biomedcentral.com/1471-2334/12/286/prepub
